# Patient and Clinician Attitudes Toward Mobile Health Apps: Qualitative Study

**DOI:** 10.2196/77519

**Published:** 2026-03-02

**Authors:** Cornelius A James, Sarah L Krein, Sarah Yon, Michael P Dorsch, John D Piette, Brahmajee K Nallamothu

**Affiliations:** 1 Department of Internal Medicine, University of Michigan, 2800 Plymouth Road, Bldg. 15, Rm 430W, Ann Arbor, MI, 48109, United States, 1 7349365216, 1 7346155845; 2 Department of Pediatrics, University of Michigan, Ann Arbor, MI, United States; 3 Department of Learning Health Sciences, University of Michigan, Ann Arbor, MI, United States; 4 Department of Veterans Affairs Center for Clinical Management Research, Ann Arbor, MI, United States; 5 Department of Behavioral and Health Education, University of Michigan, Ann Arbor, MI, United States; 6 Department of Clinical Pharmacy, University of Michigan, Ann Arbor, United States

**Keywords:** mobile health, mHealth, digital health technology, mhealth apps, health apps, qualitative

## Abstract

**Background:**

Mobile health (mHealth) apps are widely available, and some have proven safe and effective for management of specific chronic conditions. Despite a high degree of interest, the potential of these technologies has yet to be realized. Patient and clinician attitudes are key factors that influence the adoption of mHealth apps but remain poorly understood, particularly in the United States.

**Objective:**

This study aimed to identify both patient and clinician attitudes that can influence recommending and adopting mHealth apps.

**Methods:**

Using well-established technology adoption and implementation science frameworks, this study included a deductive content analysis using a rapid qualitative analytic method. Semistructured interviews were conducted with patients and clinicians to identify technical and material, social and personal, and policy and organizational factors that can influence the recommendation or adoption of mHealth apps. The interviews and data analysis were performed between September 2023 and August 2024.

**Results:**

Participants included 20 clinicians (n=12, 60% general internists) with a mean time in practice of 17 (SD 11.6) years, and 28 patients with a mean age of 59 (SD 12.1) years. A total of 7 categories related to patients’ and clinicians’ attitudes toward mHealth apps emerged: (1) apps as tools to improve health by extending care, (2) the role of apps in enhancing the patient–clinician relationship, (3) the need for simplicity and efficiency in app design, (4) the influence of prior experience with mHealth apps, (5) comfort with technology, (6) recommendations from trusted sources, and (7) education and hands-on experience. Although similar factors were considered by patients and clinicians, their views about older adults’ interest and ability to use mHealth apps differed.

**Conclusions:**

Understanding patient and clinician views about mHealth apps provides critical insights for developing approaches to facilitate their use. These findings suggest patients and clinicians share similar views about the benefits of mHealth apps. Nonetheless, clinicians’ perceptions about older patients’ interest and ability to use mHealth apps may negatively impact recommendation of mHealth apps and subsequent adoption by older adults.

## Introduction

Mobile health (mHealth) apps can collect health information to facilitate monitoring of patients’ health and behaviors throughout the day and provide real-time, personalized recommendations at key decision points (eg, when making dietary decisions). mHealth apps can increase physical activity [1] and address conditions such as depression, anxiety [2], tobacco use [3], and alcohol use disorder [4].

There are over 350,000 mHealth apps available in app stores [5], and nearly two-thirds of US adults report use of an app for health-related purposes [6]. The global mHealth market was valued at US $83.17 billion in 2023 and is expected to grow to US $249.05 billion in 2032 [7]. Despite enthusiasm for app use, adoption has lagged in underserved populations and among persons with chronic conditions [8,9]. With an increasing life expectancy [10], increased burden of chronic diseases in the United States [11], and persisting barriers to health care access [12], mHealth apps have the potential to improve health outcomes by offering the convenience of readily available health care solutions.

Getting safe and effective apps into the hands of people who need them most, and sustaining use of these apps remains a challenge. For instance, older aged persons, women, and those with lower educational attainment are less likely to use apps, and the reasons for these disparities are unknown [13]. Additionally, it has been estimated that approximately 71% of app users discontinue use within about 90 days [14]. Further, many clinicians either recommend mHealth apps inconsistently or they do not recommend them at all [15-17].

There are technology level, individual level (eg, patient and clinician), organization level, and policy level factors that may impact the adoption of mHealth apps, with each of these factors influencing the other(s) to varying degrees [18-21]. Studies have shown that patient and clinician attitudes about mHealth apps are amongst the most influential factors that determine mHealth app use [18,21,22]. In a study by Vo et al [23], patients identified patient engagement and patient empowerment as potential strengths of mHealth apps, and trustworthiness, limited personalization (eg, relevance to their condition, culture, and accessibility) as potential weaknesses. Sarradon-Eck and colleagues [24] showed that general practitioners in France had mixed views about mHealth apps, with some adopting a favorable view and others expressing skepticism or resistance. As an example, the main perceived benefit expressed by clinicians was related to the apps’ potential to facilitate completion of their work and follow-up care because of the reliable and objective measurements provided by apps. Clinicians have also expressed concerns about mHealth apps negatively impacting the patient–clinician relationship because they could lead to decreased face-to-face interactions [25]. Additionally, in a study by Dahlhausen and colleagues [26], clinicians identified their ability to effectively advise patients on their use of mHealth apps, insufficient evidence supporting use and liability as concerns.

A small number of studies have explored the views of both patients and clinicians simultaneously. For example, Byambasuren and colleagues [27] conducted semistructured interviews of general practitioners and patients to identify determinants of prescribing mHealth apps in Australian general practice settings. General practitioners identified generational differences, limited knowledge of apps that can be prescribed and trustworthy sources to access them, time required to learn to use apps, concerns about privacy, and safety and trustworthiness as potential barriers. The main barriers identified by patients were older age and usability of mHealth apps. Importantly, patients did not share clinicians’ concern about data privacy and data safety, highlighting an opportunity to develop a shared understanding of these potential barriers. Jezrawi et al [28] conducted surveys of both patients and physicians, as well as focus groups with patients to understand their attitudes about mHealth apps. Physicians who prescribed apps to patients relied most on personal opinions and recommendations from patients, whereas recommendations from trusted medical organizations or doctors were the most frequently cited sources that patients considered when making decisions about an mHealth app.

Even fewer studies have directly compared the views of patients and clinicians. For instance, to understand patients’ and clinicians’ perceptions about mHealth apps, Liu and colleagues [29] conducted semistructured interviews with 9 patients and 14 clinicians and triangulated their data to generate 5 themes, including 1 theme indicating different concerns between the 2 groups related to mHealth apps. For example, patients were more concerned about the effectiveness of mHealth apps, and clinicians were most concerned about the usability of mHealth apps. In another study, Barber and colleagues [30] conducted semistructured interviews to elicit patients’ and physicians’ perspectives on the use of an mHealth app for management of osteoarthritis. Investigators found that patients were supportive of app use, but physicians were more skeptical.

While the United States has contributed more mHealth-related research than any other country in the world [31], most research specifically investigating patients’ and clinicians’ broader attitudes about mHealth apps is based outside of the United States [19,23,24,26,27,32-42]. This is important because non-US countries may have regulatory requirements that influence attitudes in ways that are not relevant to US consumers and clinicians [43-45]. Moreover, realizing the potential of mHealth apps to improve health outcomes requires an understanding of both the individual and shared attitudes of patients and clinicians about mHealth. This understanding may facilitate the development of interventions to foster a shared mental model about the strengths and limitations of mHealth apps to enhance shared decision-making and increase patient and clinician adoption of safe and effective mHealth apps.

The objective of this study was to investigate patient and clinician attitudes about mHealth apps and to identify factors that may influence clinicians’ willingness to recommend and patients’ willingness to use mHealth apps. For this study, conversations about mHealth apps were grounded by discussion of a specific mHealth app being evaluated in an ongoing randomized controlled trial (RCT). The ability to examine these questions during the deployment of an existing mHealth app—rather than a theoretical tool—provided us with a unique opportunity to generate novel insights.

## Methods

### Qualitative Research Approach and Paradigm

The study team included clinicians (CAJ, MPD, and BKN), individuals with expertise in qualitative methods (SLK), implementation science (SLK), health services research (BKN, SLK, and JDP), and digital health technologies (DHT) (CAJ, BKN, MPD, and JDP). All members of the study team were employed by the University of Michigan (U-M), a large midwestern public university (CAJ, SLK, MPD, JDP, BKN) or a Veteran’s Health Administration hospital (SLK, JDP). Given the qualitative nature of this study and the aim of understanding participants’ subjective experiences, a constructivist research approach was used.

### Study Design and Setting

We used a descriptive qualitative study design to examine patient and clinician experiences with mHealth apps [46]. The study occurred at U-M from September 2023 to August 2024. The study adhered to the standards for reporting qualitative research reporting guidelines [47].

### Participants and Sample

#### Patients

LowSalt4Life is a mHealth app designed to decrease dietary sodium intake in patients with hypertension. The app uses geofencing and reinforcement learning technology to deliver personalized, contextual push notifications about salt intake when patients are in grocery stores and restaurants. A pilot RCT (LowSalt4Life-1—NCT03099343) showed that contextual just-in-time notifications from the LowSalt4Life app resulted in greater reduction in dietary sodium intake in adults with hypertension compared to a control group (no app) over an 8-week period [48].

Purposive sampling methods were used to recruit patients from a cohort that participated in the LowSalt4Life-2 RCT (NCT05396001), which included 200 patients in the experimental arm (received personalized LowSalt4Life app) and 200 patients in the control arm [49]. Participants randomized to use the personalized LowSalt4Life app were invited via email to participate in a semistructured interview after exiting the LowSalt4Life-2 study.

#### Clinicians

Purposive sampling was used to recruit clinicians who provide direct care to patients with hypertension, including dietitians, pharmacists, primary care physicians, nephrologists, and cardiologists. Clinicians were contacted via the closed, moderated U-M Division of General Medicine email listserv or through targeted, direct emails to inquire about interest in participating in the study. Additionally, snowball sampling, in which interviewees were asked to identify colleagues who could provide valuable insights into the study, was used to recruit additional clinicians. These clinicians were also emailed invitations to participate in the study.

The clinician interviews preceded the patient interviews because patients were sampled from the LowSalt4Life-2 study and completion of the study was required before patients were approached for an interview.

### Data Collection

The interview guides were developed using constructs from the technology acceptance model (TAM) [50,51], the unified theory of acceptance and use of technology (UTAUT) [52,53], the theoretical domains framework [54], and the technology integration model (TIM) [55].

The patient interview guide included two domains: (1) technical and material factors and (2) social and personal factors (Multimedia Appendix 1). The clinician interview guide included three domains: (1) technical and material factors, (2) social and personal factors, and (3) policy and organizational factors (Multimedia Appendix 1). The technical and material factors domain included constructs such as perceived usefulness and perceived ease of use. Constructs in the social and personal factors domain included attitudes, self-efficacy, identity, and social influence. Finally, workflow, training, and education were the constructs included in the policy and organizational factors domain.

The patient guide included 19 questions (plus probing questions), and the clinician guide included 14 questions (plus probing questions) (supplement). Both interview guides were iteratively updated as new information was encountered during interviews, and the final guides were approved by members of the study team (CAJ, SLK, BKN, JDP, and MPD).

The interviews were conducted either virtually via the Zoom platform or by phone by a clinician (CAJ) and a nonclinician research associate (SY). Prior to interviews, clinicians viewed a brief presentation about the LowSalt4Life app to (1) facilitate a shared understanding of the general capabilities of mHealth apps, (2) evaluate their attitudes about the LowSalt4Life app, and (3) have a common reference app to allow comparison of clinician and patient attitudes about mHealth apps in general. Each interview was audio recorded and transcribed.

### Data Analysis

We conducted a deductive content analysis [56-58] using a rapid qualitative analysis approach to identify subcategories and categories within the domains of the theoretical frameworks. Rapid qualitative analysis is a particularly useful method when the data collection is targeted (eg, selection of interviewees) and processes (interview protocol) are highly structured [59-61]. The rapid qualitative analysis approach allowed us to maintain rigor while also working within the constraints of another study. Rapid qualitative analysis involves three analytic steps: (1) summarization of individual transcripts, (2) consolidation of transcript summaries by participant type (eg, patient or clinician), and (3) in-depth analysis [62].

Two study team members (CAJ, SLK) developed a summary template based on the domains in the interview guide, then independently reviewed, summarized, and discussed 3 interview transcripts to test and refine the template and summarization process. Thereafter, concurrent with ongoing data collection, each interview transcript was reviewed in detail, and transcript summaries that organized the data into the relevant domains were generated by the first author (CAJ). Data from the transcript summaries were transferred to patient or clinician data matrices to allow visualization of the data across domains, constructs, and participants. A detailed review of the data matrices by several study team members (CAJ, SLK, SY) led to the identification of patient and clinician subcategories within each domain. Through triangulation, the subcategories were then aligned to create broader categories by placing the patient and clinician data in a table that allowed comparison across domains and constructs (). CAJ and SLK met biweekly to discuss the data and resolve any discrepancies. The categories were finalized during meetings with members of the research team (CAJ, SLK, SY, JDP, and BKN).

### Ethical Considerations

The study protocol (HUM00238747) was approved by the U-M Institutional Review Board. All participants provided written informed consent. All the study data was deidentified and stored in a Health Insurance Portability and Accountability Act–compliant, U-M–approved electronic database to ensure privacy and confidentiality of participants. Patients received a US $25 gift card after completing the interview. Clinicians did not receive an incentive for participating in the study.

## Results

### Patient and Clinician Information

Prior to initiating the study, we planned to interview 20 clinicians and 20 patients. However, data saturation (no new analytical information identified by the research team) was reached after 20 clinician interviews and 28 patient interviews. Among the 20 clinicians interviewed, the mean number of years in practice was 17 (SD 11.6) years (Table 1) and most were general internists (n=12, 60%). The clinician interviews lasted between 22 to 49 minutes. The mean age of the 28 interviewed patients was 59 (SD 12.1) years, and 16 (57.1%) patients were female (Table 1). Two (7.1%) patients self-identified as Asian, 4 (14.2%) self-identified as Black/African American, 21 (75%) self-identified as White, 1 (3.6%) self-identified as unknown, and none self-identified as Hispanic. The patient interviews lasted between 21 and 63 minutes.

**Table 1.** Patient and clinician demographic data.

Characteristics	Participants
Patients (n=28)	
Age (y), mean (SD)	59 (12.1)
Sex, n (%)	
Female	16 (57.1)
Male	12 (42.8)
Race or ethnicity,^a^ n (%)	
Asian	2 (7.1)
Black/African American	4 (14.2)
Hispanic	0 (0)
White	21 (75)
Unknown	1 (3.6)
Education, n (%)	
High school graduate/GED^b^	1 (3.6)
2 y degree/some college	1 (3.6)
Bachelor’s degree	12 (42.8)
Graduate degree and/or professional degree	14 (50)
Clinicians (n=20)	
Sex, n (%)	
Female	13 (65)
Male	7 (35)
Specialty, n (%)	
Cardiology	2 (10)
General medicine	12 (60)
Nephrology	3 (15)
Nutrition	1 (5)
Pharmacy	2 (10)

a Race or ethnicity was self-reported.

b GED: general educational development.

Patient and clinician attitudes about mHealth apps fit within 1 of 7 categories related to the 3 theory-based domains (Table 2). Within the technical and material factors domain, the categories identified were apps as tools to improve health by extending care, the role of apps in enhancing the patient–clinician relationship, and the need for simplicity and efficiency in app design. Categories in the social and personal factors domain included prior experience with mHealth apps, comfort with technology, and the influence of recommendations from trusted sources. Education and hands-on experience were the category identified in the policy and organizational factors domain. These categories are described in more detail below.

**Table 2.** Categories and subcategories related to patient and clinician attitudes about mHealth^a^ apps.

Domain and category	Patient subcategory	Clinician subcategory
Technical and material factors		
Tools to improve health by extending care	mHealth apps are tools to improve health	mHealth apps can extend care beyond episodic visits
Enhancing the patient–clinician relationship	mHealth apps can improve patient–clinician engagement	mHealth apps can improve the patient–clinician relationship, if beneficial
Simplicity and efficiency	mHealth apps must be simple	Patients stop using mHealth apps that are complex or require a significant time commitment.
Social and personal factors		
Prior experience with mHealth apps	Positive experience with mHealth apps but concerns about data privacy	Personal use and prior recommendation of mHealth apps, but concerns about data privacy
Comfort with technology	Confident when using technology	Confident when using and discussing technology; older adults are not confident when using technology
Recommendations from trusted sources	Influence of trusted individuals	Influence of trusted organizations and colleagues
Policy and organizational factors		
Education and hands-on experience	Access to education about an mHealth app	Use of mHealth app prior to recommending

a mHealth: mobile health.

### Technical and Material Factors

#### Tools to Improve Health by Extending Care

Both patients and clinicians described 1 of the benefits of mHealth apps as the capacity for use outside traditional health care settings. For patients, mHealth apps provide them with an always accessible tool to encourage healthier behaviors.

*So, just that tool to have with you, you know an app on your phone. It’s very helpful to be able to have that information with you.*

[Patient 27, female, 55-year-old]

For clinicians, mHealth apps can reinforce their health-related recommendations outside of regularly scheduled clinic visits.

*This is almost a way to extend my advice in a more practical way that allows them to be more aware and hopefully more compliant, or make better choices...*

[Clinician 8; 19 years of experience]

#### Enhancing the Patient–Clinician Relationship

In general, patients and clinicians believed that mHealth apps could improve the patient–clinician relationship. Patients perceived that accurate, organized data from mHealth apps could improve their engagement with their health care, including providing additional information to facilitate meaningful conversations with clinicians and changes in management.

*Yeah, I think it’d be terrific for my provider to be able to see the data...I think that that could be helpful to fuel a good conversation with a doctor.*

[Patient 23, male, 57-year-old]

Clinicians suggested that equipping patients with a tool to support their health care would enhance their relationships with patients.

*So, either way it would augment the physician-patient relationship. So even if it [patient data] does not feed to me, I think it would still allow me to give a patient a tool that can help them with their, with supporting their medical needs.*

[Clinician 8; 19 years of experience]

While clinicians recognized the potential benefits of mHealth apps on the patient–clinician relationship, they expressed concerns about remembering available apps, a need for support from staff, and managing new data streams.

*So, incorporation of this [mHealth app] into practice would involve others in the practice to assist. Either nurse practitioner or preferably a dietitian.*

Clinician 18; 44 years of experience]

*On the clinician perspective, okay, now that you have all this extra data...Do you have the time to take a look at it. And then how do you actually turn that into changes in your management?*

[Clinician 17; 8 years of experience]

#### Simplicity and Efficiency

Patients identified being easy to use and requiring a low time commitment as key considerations for the use of mHealth apps. In fact, they highlighted that complexity could be an important barrier.

*Well, if it got too techy, I wouldn’t have the patience... If it gets too technical, then no, it’s not going to be useful or helpful to me.*

[Patient 9, female, 86-year-old]

Additionally, clinicians expressed concerns about patients starting to use apps but eventually discontinuing use for reasons including complexity, too much of a time commitment, or too much manual data entry.

*Have to keep an eye on the utilization. I think if they find it hard to use, they won’t use it, or they might use it initially, but then fall off.*

[Clinician 18; 44 years of experience]

*If you’re having the data, enter everything and it’s not helping you along the way gets tiresome to patients. So, they usually use it for a while and give up.*

[Clinician 16; 28 years of experience]

### Social and Personal Factors

#### Prior Experience With mHealth Apps

Patients and clinicians both talked about positive experiences with mHealth apps. Many used apps for fitness tracking, weight loss, and sleep monitoring. For example:

*I have apps that are really important to me, and one of them is my EKG app.*

[Patient 3, female, 67-year-old]

*I mean, I think I love mobile health applications. I mean, I have a ton of them on my phone for all kinds of different things.*

[Clinician 9; 1 year of experience]

However, both patients and clinicians expressed concerns about data privacy, with some patients stating that they would be unwilling to use an mHealth app that tracked specific types of data (eg, location).

*The part that I think would be almost a deal breaker for me is the like background location tracking, or like you have it on all the time...I just would have some like privacy concerns.*

[Patient 16, male, 34-year-old]

*And so, you know, some folks will probably have a hard time with the kind of knowledge that they are quote unquote being tracked.*

[Clinician 17; 8 years of experience]

#### Comfort With Technology

Confidence and comfort with using technology were common considerations in the use of mHealth apps for both patients and clinicians. Patients, for the most part, expressed confidence when using various types of technology and generally described themselves as tech-savvy. Older patients noted that technologies should be designed to facilitate use by older adults and expressed a willingness to learn to use health technologies if necessary.

*I am the type where if there is a need to use an app, especially for healthcare I would learn to use the app.*

[Patient 8, male, 72-year-old]

*There are some things that I can do on a computer, some things that I can do on my tablet. And if I learn to do something, I pretty much know it.*

[Patient 9, female, 86-year-old]

Clinicians also described being open to new technologies and viewed themselves as tech-savvy. In addition, they expressed confidence when discussing technologies with patients.

*Yeah, as long as I’ve had some time to work with it, I can. You know I’m reasonably confident. But I’m not. I don’t have a phobia of technology. I am open to it.*

[Clinician 18; 44 years of experience]

On the other hand, clinicians generally did not view patients as tech-savvy, and they specifically expressed concerns related to older adults’ interest and ability to effectively use mHealth apps and other technologies.

*I have some patients where I could probably tell you right off the bat. They’re not gonna be interested, just cause they’re not into tech type things, you know. I have some patients that still, you know, wish they could have their flip phone. And you know, some of the old school stuff or you know, patients that just prefer to stay off their phones altogether if they can. Usually, it’s my older generation of patients, although sometimes they surprise you.*

[Clinician 3; 10 years of experience]

#### Recommendations From Trusted Sources

Trusted sources were an important factor identified by both patients and clinicians related to their willingness to use or recommend mHealth apps. A recommendation from health care providers, friends, or family members would influence patients’ decisions to use an app.

*And my doctor, if she said, I want you to try this application because I think it’s gonna make an improvement of your health, then I would do it.*

[Patient 7, female, 62-year-old]

Guideline recommendations from professional societies or organizations would influence clinicians’ decisions to recommend an app to their patients. Additionally, some clinicians stated that if their colleagues were recommending an app to their patients, or if a respected colleague recommended an app, they would be more inclined to recommend the app to their own patients.

*I mean, I do think something like, you know, a major guideline recommendation would be at least something, particularly if you know I’m reading the recommendation, and I see there is something behind it. Potentially like a provider that I like, or trust would be useful too.*

[Clinician 1; 15 years of experience]

### Policy and Organizational Factors

#### Education and Hands-on Experience

Another facilitating factor for use of mHealth apps was gaining familiarity with an app. Specifically, patients were interested in readily available educational resources or assistance to increase their comfort with an app and to troubleshoot any problems that arise.

*Maybe like something where you could have a chat session with somebody to help you get the answer you need.*

[Patient 4, male, 57-year-old]

Most clinicians preferred to personally use an app prior to recommending it to patients. Additionally, many clinicians described recommending mHealth apps that they had personally used.

*I think just seeing the like interfaces on the app, seeing what the patient sees is huge for me. I don’t like to like throw them blind into something.*

[Clinician 4; 2 years of experience]

## Discussion

### Principle Findings

mHealth apps are an increasingly important part of the health care landscape and can enhance patients’ autonomy and engagement with their health care. Our findings show that overall, patients and clinicians have positive attitudes about mHealth apps, including their potential usefulness in extending care outside of traditional health care settings and enhancing the patient–clinician relationship. Patients and clinicians identified several common factors to facilitate the recommendation or adoption of mHealth apps. These factors included the importance of the ease of use of an app, receiving recommendations about mHealth apps from trusted sources, and access to education about or hands-on experience with mHealth apps. Prior experience with mHealth apps and comfort using technology were also described as facilitating factors by both patients and clinicians.

Other studies have indicated that both patients and clinicians have concerns about mHealth apps eroding the patient–clinician relationship and therapeutic alliances because of less face-to-face interaction [63]. This concern is well-founded as the introduction of technology can lead to a form of automation bias in which clinicians over-rely on mHealth apps and their outputs, or inappropriate delegation of tasks to mHealth apps [64]. Clinicians will need to exercise vigilance and make nuanced decisions about the appropriateness of mHealth apps for a given task to both ensure safety and that the patient–clinician relationship remains intact. Further, measures to avoid patient overreliance on mHealth apps will need to be in place because this could also negatively impact the patient–clinician relationship [65]. In a study by Preiser and colleagues [66], general practitioners in Germany noted that a symptom checker app could result in decreased unnecessary office visits and decreased clinician workload. However, the clinicians preferred that patients err on the side of caution and seek their help early to circumvent avoidable suffering. Ultimately, technology and safe, humane care should not be treated as mutually exclusive. Consistent with the TIM [55], this will require further exploration of how mHealth apps can extend patient and clinician capabilities, and both patients and clinicians acknowledging the strengths and limitations of technology and one another [67].

While mass media campaigns [68] and direct-to-consumer marketing [69] have proven effective at influencing patient health decisions, patients in our study noted that their attitudes about an app would be most influenced by trusted individuals (eg, friends, family, and health care providers). Therefore, clinicians must actively engage with patients and their family, friends, and caregivers to encourage the adoption and sustained use of safe and effective mHealth apps [70]. Further, studies have shown that patients’ adoption of health-promoting behaviors may be influenced by individuals in nontraditional health care settings. For example, in an RCT by Victor and colleagues [71], Black male patients with uncontrolled hypertension were assigned to either an intervention group in which barbers at Black-owned barbershops encouraged meetings in barbershops with specialty-trained pharmacists who prescribed antihypertensive medication, or to a control group in which the barbers encouraged lifestyle modifications and doctor appointments. While both groups experienced improvement in their blood pressure at 6 months, the improvement was much greater in the group that met with the pharmacists and were prescribed antihypertensives. This study highlights both the benefits of treating patients in their natural environments and the benefits of leveraging the influence of trusted members of the community to promote healthy behaviors. Partnerships such as this should be explored to promote the adoption and sustained use of mHealth apps that have proven safe and effective.

Clinicians in our study noted that trusted colleagues, specialty organizations, and evidence-based guidelines, each of which may be related to the subjective norms described by UTAUT, would strongly influence their decision to recommend an app to a patient. However, clinicians did not describe app certification or approval by a regulatory agency (eg, Food and Drug Administration [FDA]) as necessary for recommending an app to a patient. In other studies, clinicians have identified a lack of certification as a barrier to recommending mHealth apps. For instance, in a study by Schroeder et al [36], clinicians expressed that they view mHealth apps similarly to other medical products and that apps should be given an official certification to ensure more trust. This study was conducted in Germany, which in 2020 became the first country in the world to approve certain mHealth apps for prescription and coverage by health insurers [26]. Additionally, Germany maintains a directory of approved mHealth apps managed by the German Federal Institute for Drugs and Medical Devices known as Digitale Gesundheitsanwendungen. As of July 2024, there were 56 mHealth apps listed by the German Federal Institute for Drugs and Medical Devices, and over 274,377 prescriptions were recorded from 2020‐2023, with Zanadio (Sidekick Health Germany), a weight loss app, prescribed most frequently during that period [72]. Another study based in the United Kingdom showed that clinicians believed the most important factor encouraging mHealth app prescription was if it had a National Health Service stamp of approval [32]. The US FDA plays a role in the regulation of mHealth apps [43] but the vast majority of mHealth apps are not regulated by the FDA. While it may not be feasible for the FDA to rigorously evaluate every app, attention should be given to developing interventions that facilitate clinician confidence in the safety and efficacy of evidence-based mHealth apps as studies have shown that many apps in app stores with high ratings often have low usability or clinical usefulness [73].

Our study showed that patients and clinicians have differing beliefs about patients’ level of tech savviness. While most patients reported using mHealth apps, clinicians generally believed that older adults would not be interested, or they would be unable to effectively use mHealth apps [63]. A recent study showed that over 81% of older adults use DHTs, and over 40% reported any use of mHealth apps [74]. Our findings suggest that clinician bias could be a barrier to recommending mHealth apps to those who may benefit most (eg, older adults). During the COVID-19 pandemic, the use of DHTs significantly increased across all age groups. However, disparities widened with older adults significantly less likely to use DHTs compared to younger adults [75]. Still, older adults’ increased use of DHTs during the COVID-19 pandemic suggests that they are interested and capable of using mHealth apps, but prior to the pandemic, they were not using them for various reasons that should be explored. Moreover, views expressed by older adults in our study are consistent with this as a 72-year-old patient stated: “I am the type where if there is a need to use an app, especially for healthcare I would learn to use the app.” Older adults are likely to have a greater burden of chronic disease [11] and impairments (eg, physical and cognitive) that can limit their ability to use DHTs, and this should be considered when recommending mHealth apps to patients. The results from our study suggest that clinicians should take an individualized approach to recommending mHealth apps to older adults. Notably, age moderates the constructs in the UTAUT framework (eg, performance expectancy, effort expectancy, social influence, and facilitating conditions) that predict behavior intention to use technologies [52,53]. When studying older adults’ use of mHealth apps, using a framework that does not include age, but does include variables associated with the aging process (eg, memory or physical limitations) may add more nuance and avoid introducing age-related bias. Further, interventions are needed to both address clinician bias [76] and to encourage patients and their families or caregivers to advocate for themselves.

Clinicians also noted barriers to recommending mHealth apps that included getting patients oriented, time constraints, a need for support from staff, and a lack of optimized workflows to facilitate review and use of data generated by mHealth apps, which highlights the importance of the environment and situational context described in the TIM [55]. These concerns must be weighed against the benefits to patients. For instance, patient-generated data has been shown to increase patient health literacy and to help patients better engage with their health providers [77,78]. User-centered mHealth apps also have the potential to increase adherence by providing individualized, just-in-time reminders and advice during vulnerable times outside of clinics and hospitals as demonstrated in conditions such as human immunodeficiency virus and congestive heart failure [79-81]. Like other studies, clinicians in our study expressed interest in having data from mHealth apps integrated into electronic health records (EHR) for efficient visualization, analysis, and application. This type of integration would require policy and organization-level interventions that support the flow of new data streams, interoperability between systems, and application of data from mHealth apps to improve patient–clinician engagement and health outcomes. Furthermore, health care organizations and app developers would need to partner with EHR vendors to ensure that their products are interoperable, allowing seamless data exchange between the EHR and mHealth apps. Collaborations between patients, clinicians, health system leaders, and developers will be necessary to ensure that apps address relevant clinical problems, are easy to use, and simple to set up. Thus, user-centered design principles addressing the patient, caregiver, and clinician perspectives must be used to facilitate recommendation by clinicians and adoption by patients.

James et al [82] described the “4E’s (ease, evidence, experience and expectations)” that inform the mindlines influencing clinicians’ decisions to recommend mHealth apps. Mindlines are collectively reinforced, internalized tacit guidelines, which are informed by brief reading, but mainly (by) clinicians’ interactions with each other (as well as) opinion leaders, patients, and pharmaceutical representatives and by other sources of largely tacit knowledge that build on their early training and their own and their colleagues’ experiences [83]. Our study reinforces the potential influence of the 4E’s described by James and raises the question of whether the mindlines concept and the 4E’s informing them could be expanded to patients. However, it should be noted that a category related to expectations did not emerge in our study, which could be due to the theoretical frameworks used and the constructs used. Therefore, the role of patients’ and clinicians’ expectations around mHealth apps should be explored.

### Limitations

Strengths of this study include using an mHealth app that was being actively studied and deployed as a common frame of reference for discussions about mHealth apps with patients and clinicians. Additionally, we combined constructs from well-established technology adoption (eg, TAM and UTAUT), and implementation science (eg, theoretical domains framework and TIM) frameworks to account for limitations of each framework, which allowed us to identify facilitators and barriers that could inform development of interventions to increase recommendation and adoption of mHealth apps by patients and clinicians.

This study also had key limitations. First, it occurred at a single academic health center in a metropolitan area. There are known geographic differences in smartphone, internet, and DHT use, with lower rates of access among patients in rural areas. Second, our study only included patients enrolled in the LowSalt4Life-2 study, a study of a single mHealth app. Hence, the results may be affected by selection bias as participants may have had a favorable view of mHealth apps and interest as indicated by their participation in the study. Third, the qualitative design of our study does not allow us to infer that the attitudes expressed by participants in our study are shared by others. Fourth, because we used a deductive analytic approach that included domains from established frameworks, we may not have identified additional relevant or novel attitudes that may have been discovered with an inductive approach. Finally, we cannot conclude that addressing the identified patient and clinician attitudes will result in increased recommendation or adoption of mHealth apps. Our study did not measure intention to use, behavior intention, use behavior, or technology use, which are downstream constructs in the TAM, UTAUT, and TIM frameworks, respectively [50-53,55]. Thus, studies are needed to determine the impact of interventions that address the identified attitudes on patient adoption and clinician recommendation of mHealth apps.

### Conclusions

In this qualitative study, patients and clinicians generally had a positive view of the potential benefits of mHealth apps. Both groups also identified several similar factors that facilitate the use of mHealth apps. However, overall, patients’ and clinicians’ views about older patients’ interest in and ability to use technology differed. As mHealth apps are developed and implemented, careful consideration must be given to both patients’ and clinicians’ attitudes and agreement and disagreement between these key stakeholders. This approach will allow the development of implementation interventions that facilitate recommendation of mHealth apps by clinicians and adoption of mHealth apps by patients.

## Supplementary material

10.2196/77519 Multimedia Appendix 1 Patient and clinician interview guides.

## Abbreviations

DHT: digital health technology
EHR: electronic health record
FDA: Food and Drug Administration
mHealth: mobile health
RCT: randomized controlled trial
TAM: technology acceptance model
TIM: technology integration model
U-M: University of Michigan
UTAUT: unified theory of acceptance and use of technology
