# Supplementary material for: Patient and Clinician Attitudes Toward Mobile Health Apps: Qualitative Study
Source: JMIR Mhealth Uhealth. 2026 Mar 2;14:e77519. doi: 10.2196/77519 (PMC12954712; doi:10.2196/77519)
Supplement: Multimedia Appendix 1 [file mhealth-v14-e77519-s001.docx]

**LowSalt4Life Patient Interview Guide (HUM00238747)**

**Date/Time:**

**Interviewer(s):**

**Respondent ID/Interview Number:**

**App + JITAI/App + Optimized JITAI:**

**Introduction**

Hello, I am [your name] from the University of Michigan (U-M) and I am working with the LowSalt4Life study team. The purpose of this interview is to help us to learn about how patients think about the use of mobile health technologies. Ultimately, we are interested in developing a mobile health (mHealth) intervention that patients with hypertension are comfortable using to improve their sodium intake, lower their blood pressure and decrease their risk of cardiovascular events. We are particularly interested in your attitudes and beliefs related to a dietary sodium just-in-time intervention (JITAI) designed to assist patients with making healthy dietary sodium choices.

In addition to patients, we are also interviewing clinicians to understand their attitudes and beliefs as they may recommend this technology to their patients.

The interview will last under an hour.

Prior to beginning the interview, we would like to get your permission to conduct this interview and record it to facilitate later transcription and coding. This study has been approved by the U-M Institutional Review Board (IRB).

Please be aware that you may ask the voice recorder to be turned off, decline to answer any interview questions, or stop the interview at any time.

All audio, notes, and transcripts will remain confidential and only be accessible to project staff. Any personal identifying information that you reveal during the interview will be removed during transcription of the interview, and all interview recordings will be destroyed after the study has been completed.

Do you have any questions for me about the study, this interview, or the consent process?

Okay. I am going to turn the recorder on now and ask for your consent so that we have it on record.

**TURN ON RECORDER**

For the record, do we have your permission to conduct and audio record this interview?

[If yes, continue to interview guide]

[If no, turn recorder off]

**BACKGROUND**

To get started, can you please tell us about your background?

**Probes:**

1. How long have you had hypertension?
2. Do you take blood pressure medications?
3. What has made managing your blood pressure difficult?
4. What has been most helpful to manage your blood pressure?

| **Technical and Material Factors** |
| --- |
| Construct/Concept: Perceived Usefulness |
| 1. **How did you benefit from use of the dietary sodium just-in-time adaptive intervention (JITAI)?**   **Probes:**   - What do you like about the dietary sodium JITAI? |
| 1. **How did the dietary sodium JITAI help you to achieve lower salt intake? Blood pressure control?**   **Probes:**   - Are there features of the dietary sodium JITAI that you found useful? - Are there features of the dietary sodium JITAI you found less useful (or a hinderance)? |
| Construct/Concept: Perceived Ease of Use |
| 1. **How did you incorporate the dietary sodium JITAI into your lifestyle?**   **Probes:**   - In what situations did you find the dietary sodium JITAI easiest to use? - How are you currently tracking your sodium, blood pressure and reporting it to your healthcare provider? |
| 1. **What were barriers/challenges to consistently using the dietary sodium JITAI?**   **Probes:**   - What features of the dietary sodium JITAI made it difficult to use? - What aspect of your lifestyle would make use of the dietary sodium JITAI difficult? - How did you feel about the notifications that you received?   - Content? Frequency? - What are your thoughts about the advice provided by the applicication? |
| 1. **What factors made it more likely that you would use the dietary sodium JITAI?**   **Probes:**   - What types of messages did you find most helpful? - When were you most likely to use the application? - What features would you add to the dietary sodium JITAI? |
| **Social and Personal Factors** |
| Construct/Concept: Attitudes |
| 1. **What is your view of technology in healthcare?**   **Probes:**   - How has technology helped your healthcare? - How has technology limited your healthcare? - Do you use any other mobile health applications? |
| 1. **What would influence your decision to use a mobile health application?**   **Probes:**   - Why did you previously (or why do you currently) use mobile health applications?   - What were/are your goals when using mobile health applications previously?   - What would influence your decision not to use a mobile health application? |
| 1. **What is your view of the dietary sodium JITAI?**   **Probes:**   - What do you like about the dietary sodium JITAI? - Is there anything that you like/dislike about the dietary sodium JITAI? |
| Construct/Concept: Emotions |
| 1. **How do you feel about using the dietary sodium JITAI?**   **Probes:**   - How did the dietary sodium JITAI impact you personally? - What feelings come to mind when you think about use of the dietary sodium JITAI? |
| Construct/Concept: Self-efficacy, experience, skills, abilities |
| 1. **How confident are you when using technology?**   **Probes:**   - On a scale of 0 – 10 (0 = not at all, 10 = very) how would you rate your tech savviness? - Are you generally an early adopter or late adopter of new technologies? |
| 1. **Do you feel confident in your ability to effectively use the dietary sodium JITAI?**   **Probes:**   - What would you want to know about the dietary sodium JITAI? |
| Construct/Concept: Identity |
| 1. **Would the dietary sodium JITAI impact your relationship with your healthcare provider(s)?**   **Probes:**   - How would this technology change what you do as a patient? - How would this technology change what your healthcare providers do? |
| Construct/Concept: Social Influence |
| 1. **Is there someone that you look up to/trust that would strongly influence your decision to use the dietary sodium JITAI?**   **Probes:**   - Your healthcare providers - Friends, colleagues, specialists, experts - Organizations, agencies |
| Construct/Concept: Training and Education |
| 1. **What additional training/education about dietary sodium JITAI would you want?**   **Probes:**   - How much would you need to know about the dietary sodium JITAI to confidently use it? |
| Misc.: AI vs Human Generated Notifications (do not inform of source of statement) |
| 1. **Which statement are you most likely to respond to positively?**  - Having high blood pressure (hypertension) can increase the chance of having heart disease or a stroke. Lowering your salt will keep your blood pressure at a healthy level (human).   Or   - When you have high blood pressure, also known as hypertension, it can increase your risk of developing heart disease or having a stroke. By reducing the amount of salt you consume, you can keep your blood pressure within a healthy range (AI). |
| 1. **Which statement are you most likely to respond to positively?**  - Instead of relying on salt, you can enhance the flavor of your food by using spices like herbs, lemon juice, garlic, olive oil, nut oil, or different types of vinegars. These alternatives can add delicious taste to your meals without relying on excessive salt (AI).   Or   - Instead of using salt, try seasoning your food with spices, herbs, lemon juice, garlic, olive oil, nut oil, or a variety of vinegars (human). |
| 1. **Which statement are you most likely to respond to positively?**  - Even if you don’t experience any noticeable symptoms, having high blood pressure can harm your blood vessels, heart, kidneys, and even your eyes. By the time you start feeling symptoms, these issues may have already become quite serious. However, you can take a proactive step towards maintaining your health by reducing your salt intake. It can contribute to keeping you healthy and prevent these problems from oucurring (AI).   Or   - You may feel fine, but high BP can damage your blood vessels, heart, kidneys, and even your eyes. By the time you notice symptoms, these problems may be very serious. Cutting back on salt can help you stay healthy (human). |
| 1. **Which statement are you most likely to respond to positively?**  - Lowering your salt can also help you cut calories. Eating less processed food is a great way to start (human)   Or   - Lowering salt intake helps cut calories. Start by eating less processed food. Its an easy way to improve your health (AI). |
| 1. **Which statement are you most likely to respond to positively?**  - Go light on dressings and sauces, which often have a lot of salt. Try skipping them or asking for them on the side (human).   Or   - When it comes to dressings and sauces, it’s best to go easy on them because they often contain a lot of salt. You can try skipping them altogether or asking for them on the side. This way, you have control over how much you use, helping you reduce your salt intake and make healthier choices (AI). |

**CONCLUSION**

Thank you very much for taking the time to participate in this interview. We greatly appreciate you making the effort as we know that your time is very valuable.

**LowSalt4Life Clinician Interview Guide (HUM00238747)**

**Date/Time:**

**Clinician title:**

**Interviewer(s):**

**Respondent ID/Interview number:**

**Site:**

**Introduction**

Hello, I am [your name] from the University of Michigan (U-M) and I am working with the LowSalt4Life study team. The purpose of this interview is to help us to learn about how clinicians like you think about the use of mobile health technologies. Ultimately, we are interested in developing a mobile health (mHealth) intervention that clinicians are comfortable recommending and/or prescribing to their patients with hypertension to improve sodium intake, lower their blood pressure and decrease their risk of cardiovascular events. We are particularly interested in your attitudes and beliefs related to a dietary sodium just-in-time intervention (JITAI) designed to assist patients with making healthy dietary sodium choices.

In addition to clinicians, we are also interviewing patients to understand their attitudes and beliefs as they will be the ones engaging with this technology daily.

The interview will last under an hour.

Prior to beginning the interview, we would like to get your permission to conduct this interview and record it to facilitate later transcription and coding. This study has been approved by the U-M Institutional Review Board (IRB).

Please be aware that you may ask the voice recorder to be turned off, decline to answer any interview questions, or stop the interview at any time.

All audio, notes, and transcripts will remain confidential and only be accessible to project staff. Any personal identifying information that you reveal during the interview will be removed during transcription of the interview, and all interview recordings will be destroyed after the study has been completed.

Do you have any questions for me about the study, this interview, or the consent process?

Okay. I am going to turn the recorder on now and ask for your consent so that we have it on record.

**TURN ON RECORDER**

For the record, do we have your permission to conduct and audio record this interview?

[If yes, continue to interview guide]

[If no, turn recorder off]

**BACKGROUND**

To get started, can you please tell us about your medical background?

**Probes:**

a. Specialty?

b. Clinical focus within specialty?

c. Years of practice?

d. Type of practice?

e. Any additional responsibilities such as research, teaching, or additional clinical roles?

| **Technical and Material Factors** |
| --- |
| Construct/Concept: Perceived Usefulness |
| 1. **How do you see patients using the dietary sodium just-in-time adaptive intervention (JITAI)?**   **Probes:**   - What patients do you see using/benefiting from use of the dietary sodium JITAI? - Generally, are your patients “tech savvy?” |
| 1. **How can the dietary sodium JITAI assist you with helping your patients achieve better blood pressure control?**   **Probes:**   - Are there features of the dietary sodium JITAI that appear to be useful? - Are there features of the dietary sodium JITAI that appear to be less useful (or a hinderance)? |
| Construct/Concept: Perceived Ease of Use |
| 1. **Which patients would you see consistently/effectively using the dietary sodium JITAI?**   **Probes:**   - To whom would you recommend the dietary sodium JITAI? - What features of the dietary sodium JITAI would make it difficult to use? - Which patients would consistently use the dietary sodium JITAI? |
| Construct/Concept: Facilitating Condition |
| 1. **What factors would facilitate recommending the dietary sodium JITAI to your patients?**   **Probes:**   - If insurance providers (payers) covered patient use of the dietary sodium JITAI, would you prescribe it for your patients? - Will financial (or other) incentives influence your decision to recommend the dietary sodium JITAI to your patients? |
| **Policy and Organizational Factors** |
| Construct/Concept: Workflow Related |
| 1. **How would the dietary sodium JITAI fit into your current clinical workflow?**   **Probes:**   - What happens during a typical visit focused on hypertension? - How are your patients tracking their sodium, blood pressure and reporting it to you currently? |
| 1. **What are barriers/challenges to incorporating the dietary sodium JITAI into your clinical practice?**   **Probes:**   - Would you need any support to successfully implement this app into your clinical practice? - What would need to happen for you to successfully incorporate the dietary sodium JITAI into your practice? |
| Construct/Concept: Training and Education |
| 1. **What training/education would you require prior to recommending use of the dietary sodium JITAI by your patients?**   **Probes:**   - How much would you need to know about the dietary sodium JITAI before confidently recommending it to your patients? |
| **Social and Personal Factors** |
| Construct/Concept: Attitudes |
| 1. **How do you view mobile health (mHealth) applications?**   **Probes:**   - What are mHealth apps that you recommend to your patients? |
| 1. **What is your view of the dietary sodium JITAI?**   **Probes:**   - What do you like about the dietary sodium JITAI? - Is there anything that you dislike about the dietary sodium JITAI? - What would patients like/dislike about the dietary sodium JITAI? |
| Construct/Concept: Emotions |
| 1. **How do you feel about incorporating the dietary sodium JITAI into your clinical practice?**   **Probes:**   - What are your experiences with use of technology in health care? - How do you see tools like the dietary sodium JITAI impacting you professionally/personally? |
| Construct/Concept: Self-efficacy, experience, skills, abilities |
| 1. **How confident are you when using technology?**   **Probes:**   - On a scale of 0 – 10 (0 = not at all, 10 = very) how would you rate your tech savviness? - Are you generally an early adopter or late adopter of new technologies? |
| 1. **Would you be comfortable informing patients of the strengths and limitations of the dietary sodium JITAI?**   **Probes:**   - What should patients know about the dietary sodium JITAI? |
| Construct/Concept: Identity |
| 1. **How do you think the dietary sodium JITAI would impact the patient-clinician relationship?**   **Probes:**   - Are there tasks that you feel comfortable delegating to this technology? - How do you see this technology impacting your relationship with patients? - How would this technology change what you do as a clinician? |
| Construct/Concept: Social Influence |
| 1. **Is there someone that you look up/trust to that would strongly influence your decision to recommend the dietary sodium JITAI to your patients?**   **Probes:**   - Friends, colleagues, specialists, experts - Organizations, agencies (i.e., USPSTF) |

**CONCLUSION**

Thank you very much for taking the time to participate in this interview. We greatly appreciate you making the effort as we know that your time is very valuable.
